# Lay Midwives: On the Front Lines of the Fight Against Maternal Mortality in Rural Guatemala

**DOI:** 10.4269/ajtmh.18-0518

**Published:** 2019-02

**Authors:** Michel Juarez, Kirsten Austad, Peter Rohloff

**Affiliations:** Wuqu’ Kawoq, Maya Health Alliance, Guatemala, Central America

Doña Clarita, an indigenous Kaqchikel Maya woman who has practiced as a lay midwife for 25 years, recently took care of a home-birth patient who had failure to progress in labor and needed to be referred to the hospital. On referral of her patient, she tearfully recounted to us, “*The doctors at the hospital told my patient I was trying to kill her. You know I would never do that. Her labor was not progressing well, so I referred her to the hospital. [The hospital staff] blame me for her complication. She wanted a home delivery, I was the one who insisted that she go to the hospital. This is not fair…*”

In our work as physicians and researchers with Maya Health Alliance, a nongovernmental health-care organization working to improve rural services for Maya communities, we hear stories like that of Doña Clarita on a weekly basis. Lay midwives, who are often the only care providers in the rural communities where they live, are routinely stigmatized for and criticized by the biomedical system for not promptly detecting and referring patients to higher level care. However, as the case of Doña Clarita highlights, patients themselves strongly prefer to deliver at home and they often resist referral to the hospital even when their midwife feels it is necessary: “*She wanted a home delivery, I was the one who insisted that she go to the hospital*.”

The fact that Doña Clarita’s patient was reluctant to go to the hospital is not surprising. Indigenous Maya women experience endemic levels of disrespectful and even abusive care in public referral hospitals. This disrespectful care can sometimes be quite overt. For example, most physicians and nurses in public hospitals are not indigenous, and they may directly discriminate against indigenous patients. Similarly, most hospital staff do not speak indigenous Mayan languages, nor do hospitals have language interpreters on staff, so opportunities for misunderstandings and non-consented care abound. Finally, institutional factors, such as overcrowding and medication stockouts, all contribute to clinical uncertainly and instability that dramatically impacts patients’ experiences of the hospital environment.

Working to reduce maternal mortality is a key global health priority. This is nowhere more the case than in Guatemala, which has one of the highest rates of maternal mortality in Latin America. Guatemala is a Central American country with a large indigenous Maya population, and most of the burden of maternal mortality falls on indigenous women, who are two to three times more likely to die during childbirth. Despite extensive policy efforts by the Guatemalan government and numerous nongovernmental organizations, most of the indigenous women in rural Guatemala continue to deliver in the home, under the care of lay midwives like Doña Clarita.

High rates of maternal mortality and late-stage presentations of emergency birthing complications to emergency departments are a common source of frustration for providers working in district health centers and regional referral hospitals. Given the widespread home-birth rates, it is common for providers in these centers and hospitals to lay most of the blame on the lay midwives. For example, a medical colleague from a busy district hospital recently told us, “*If lay midwives didn’t exist, rural indigenous women would come to the hospitals to give birth. That would be the best way to decrease maternal mortality in the country*.”

However, in our view, combatting maternal mortality in Guatemala will require willingness to move past the urge to lay blame, and instead grapple seriously with the challenge posed to us by Doña Clarita’s patient. Why is it that so few indigenous women are willing to deliver in a facility, even when such a delivery is clearly indicated? And what can we do about it?

Importantly, the Guatemalan Ministry of Health increasingly recognizes the importance of these questions and is beginning to address the need to change hospital culture and clinical policies to make obstetrical care for indigenous women more respectful and less frightening. For example, the recent Ley Para la Maternidad Saludable (Healthy Maternity Law) calls for cost reforms, better access to free medications, and respect for intercultural differences. However, there is an important gap between the on-paper provisions of this law and reality, as few elements of the law have been implemented in any of Guatemala’s hospitals. For example, the law allows midwives or family members to accompany patients within the hospital environment. However, most hospitals continue to prohibit the entrance of midwives. Health-care workers say this is because they lack the resources and the hospitals are too crowded to comply with the law. “*We can’t let midwives come in with their patients because we are too crowded!*” and “*We have asked the authorities to give us more resources to give better attention, but we haven’t had anything.*”

At Maya Health Alliance, we are collaborating with a group of 41 lay midwives (∼1,000 births/year) and the regional referral hospital in the Chimaltenango department of central Guatemala, working to find answers to these important questions. Recognizing that lay midwives have little formal support and often encounter significant resistance from patients and families themselves when referrals are needed, we have begun a simple mobile phone application to guide midwives through emergency checklists and to connect them, 24-hours a day, to an on-call medical team, which can help them make emergency decisions.

We are also collaborating with the hospital to deploy a team of care navigators. Care navigators are indigenous women who received training from our staff on how to navigate emergency situations and hospital culture. When a lay midwife decides that an emergency referral is needed, she can activate a care navigator, who will then take charge of the referral. This includes working with the family to arrange emergency transportation and physically accompanying the patient to the hospital. In the hospital, the care navigators perform many services. They provide language interpretation services for the many patients who cannot speak Spanish. They are a source of emotional support, such as accompanying the patient into the operating theater and advocating to hospital staff if she feels that the patient is not receiving respectful care.

We believe that this collaborative work between community-based providers, lay midwives, and the hospital team is helping to improve the quality of obstetrical care and to reduce patients’ and midwives’ distrust of that care. Our ultimate goal is to create a more positive hospital experience for patients which, we hope, will increase future utilization of hospital services, as a woman who has a positive experience will be more likely to return in a subsequent pregnancy or recommend services to her friends. Finally, we also believe the collaboration is valuable for hospital staff morale and for creating a learning culture among providers. For example, the care navigators provide valuable language interpretation and cultural mediation services for physicians and nurses. This in turn helps to reduce their own stress and correct or eliminate misperceptions and stereotypes about indigenous patients.

Preliminary anecdotal feedback from both lay midwives and hospital staff so far has been overwhelmingly positive, and we will be conducting a formal study to confirm these anecdotes, to be published at a later date. For example, a resident physician recently told us, “*When patients do not understand Spanish, we ask the care navigator to help us translate and to explain to patients their conditions and risks*.” Another senior district physician told us, “*We would have had more maternal deaths in the area without [this project]*.” By working in a collaborative way with lay midwives and with their patients, we believe that the problem of maternal mortality in rural Guatemala can be solved. We also believe that our experience is relevant to other countries and contexts where home births are common. We urge other practitioners around the world to consider ways in which stronger, supportive ties with frontline obstetrical providers—lay midwives and community health workers—can be forged.

